# Acute necrotizing encephalopathy associated with lymphoma-associated hemophagocytic lymphohistiocytosis: A case report and literature review

**DOI:** 10.3389/fonc.2022.986957

**Published:** 2022-10-31

**Authors:** Wenqiang Sun, Changchang Fu, Xueping Zhu

**Affiliations:** Department of Neonatology, Children’s Hospital of Soochow University, Suzhou, China

**Keywords:** hemophagocytic lymphohistiocytosis, lymphoma, acute necrotizing encephalopathy, cytokines, steroids

## Abstract

Damage associated with lymphoma-associated hemophagocytic lymphohistiocytosis (LA-HLH) to the central nervous system (CNS) is not uncommon. However, the combination with brain damage resembling acute necrotizing encephalopathy (ANE) is rarely reported. Herein, we introduce the diagnosis and treatment of a case of ANE associated with LA-HLH in our hospital and review the relevant literature. After treatment, the child was discharged with only dysarthria and decreased sucking ability. The child is now discharged from the hospital for 6 months with regular follow-up. There were no disease recurrence signs. LA-HLH and ANE were related to cytokine storm. Therefore, early steroid application is essential for treating these diseases.

## Introduction

Hemophagocytic syndrome (HPS), also called hemophagocytic lymphohistiocytosis (HLH), is an inflammatory syndrome depicting excessive and abnormal T lymphocytes and mononuclear phagocyte activation. Moreover, there is a massive release of inflammatory factors accompanying hemophagocytosis of tissues and organs, including the primary and secondary categories (1). Lymphoma-associated hemophagocytic lymphohistiocytosis (LA-HLH) is a common cause of secondary HLH (2). Acute necrotizing encephalopathy (ANE) is considered one of the most critical subtypes of acute encephalopathy (3). It has a mortality rate of up to 30%, and most surviving cases suffer from moderate to severe disability. Damage associated with LA-HLH to the central nervous system (CNS) is not uncommon, but the combination with ANE is rarely reported (4–6). In this study, we reported a case of ANE associated with an LA-HLH and reviewed the relevant literature to discuss the disease’s clinical features and treatment points.

## Case description

**Figure 1:**
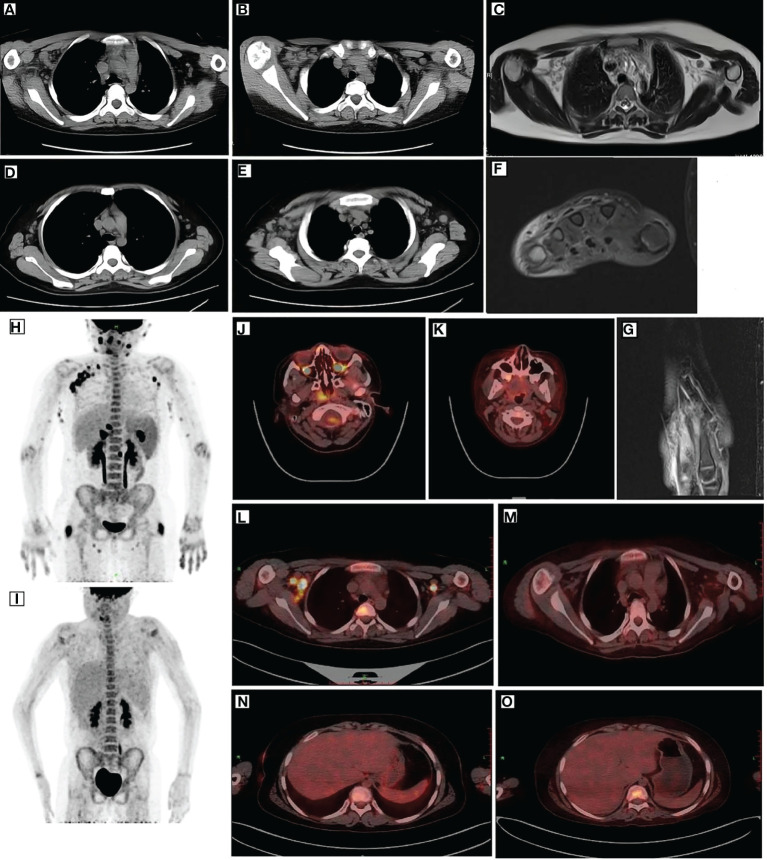
Imaging of the patient. CT **(A, B)**, MRI **(C)**, and PET **(L)** revealed multiple enlarged lymph nodes in the mediastinum and axilla with elevated glucose metabolism. After remission, CT **(D, E)** and PET **(M)** showed smaller lymph nodes than before and decreased glucose metabolism. Before treatment, PET **(H)** depicted multiple lymph node enlargements and tissue involvement with abnormally increased glucose metabolism. After remission, PET **(I)** revealed a significant reduction in the original lymph node and tissue lesions and decreased glucose metabolism. After remission, PET **(J, L)** indicated elevated FDG metabolism in the posterior nasopharyngeal wall and central bone marrow **(K, M)**, suggesting normal FDG metabolism. MRI **(F, G)** indicated edema around the dorsal extensor tendons of the 2nd to 5th metacarpals of the right hand and soft tissue swelling around the right wrist. PET **(N)** depicted increased liver volume. Moreover, repeat PET **(O)** after remission indicated increased liver volume and diffused hypodensity of the liver parenchyma. CT, computed tomography; MRI, magnetic resonance imaging; PET, positron emission tomography.

**Figure 2:**
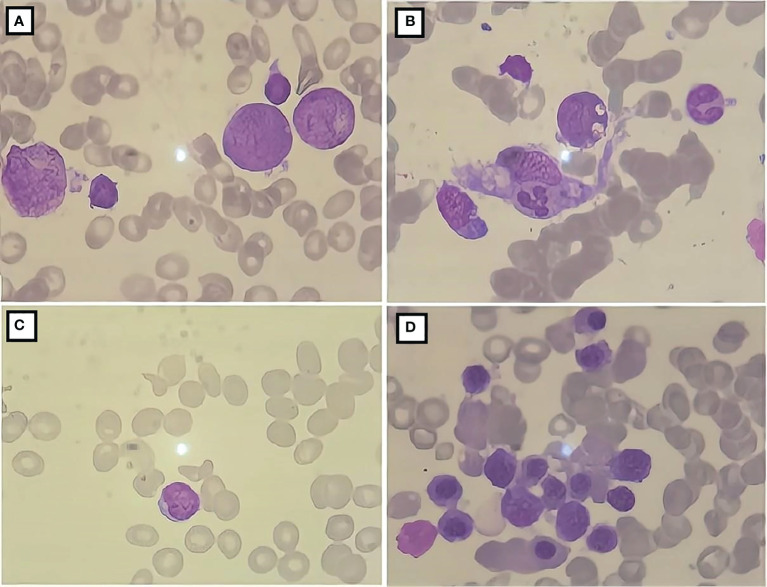
Bone marrow cell morphology of the patient. **(A)** The proliferation of bone marrow is active; **(B)** the presence of phagocytic cells; **(C)** severely diminished nucleated cell proliferation; **(D)** active bone marrow nucleated cell proliferation.

A 10-year-old female patient was admitted for the first time to our hospital due to a persistent high fever for 19 days. The girl was born to healthy, non-consanguineous parents without any family history of neurological and hematological disorders. There were no abnormalities in the patient’s birth history. She developed a persistent high fever (up to 40.2°C) for 19 days and showed no improvement after treatment with second-generation cephalosporin in other hospitals. On admission, her blood pressure was 102/68 mmHg (1 mmHg = 0.133 kPa), pulse was 102 beats/min, and body temperature was 39.3°C. She had a moderately anemic appearance with multiple enlarged lymph nodes of the neck, axilla, and groin and also had hepatosplenomegaly. Blood investigations (**Table 1**) revealed the following: decreased white blood cell count, 3.86 × 10^9^/L; percentage of lymphocytes (LY%), 36.8%; absolute neutrophil count, 1.98 × 10^9^/L; hemoglobin, 92 g/L; and fibrinogen, 2.12 g/L. PCT was significantly elevated (5.6 μg/L). Magnetic resonance imaging (MRI) and computed tomography (CT) (**Figures 1A–E**) showed multiple enlarged lymph nodes of the mediastinum and the axilla. MRI (**Figures 1F, G**) indicated edema around the dorsal extensor tendons of the 2nd to 5th metacarpals in the right hand and soft tissue swelling around the right wrist. Bone marrow cell morphology (**Figure 2A**) on the second day of admission suggested that the proliferation of bone marrow was active. Flow cytometry analyzed 10.9% of the mature lymphocyte population in the bone marrow, with 0.2% of the CD5^+^CD10^−^ mature clonal B-lymphocyte population being visible. After 8 days of anti-infection treatment with third-generation cephalosporin, the child’s fever has not improved. Blood investigations (**Table 1**) revealed the following: decreased white blood cell count, 1.23 × 10^9^/L; LY%, 49.6%; absolute neutrophil count, 1.12 × 10^9^/L; hemoglobin, 75 g/L; and fibrinogen, 1.16 g/L. In addition, natural killer (NK) cell activity was decreased. The patient had elevated triglycerides (3.21 mmol/L), lactate dehydrogenase (789 U/L), ferritin (1,102.5 pmol/L), soluble CD25 (sCD25) (1,668.8 pg/ml), interleukin-6 (IL-6) (138.2 pg/ml), and TNF-α (16.7 ng/ml). The microbiological investigations ruled out bacterial, viral, and fungal infections, including EBV. Autoantibody and antinuclear antibodies were negative. Whole-exome sequencing did not identify primary HLH-associated genes. Bone marrow cell morphology (**Figure 2B**) revealed phagocytic cells. In addition, bone marrow cell morphology revealed a small number of abnormal lymphocytes with an oval-like cell cytosol, and a few granules in the cell cytoplasm were seen. Flow cytometry analysis of 12.7% of the mature lymphocyte population in the bone marrow, with 4.8% of the CD5^+^CD10^−^ mature clonal B-lymphocyte population (FSC increased), revealed the following: expression of CD19, FMC-7, CD10, CD20, KAPPA, CD38, and CD45; weak expression of CD22; and no expression of CD4, CD8, CD3, LAMBDA, CD56, CD5, CD57, CD200, CD79b, CD23, CD103, and CD11c. The bone marrow fluorescence *in-situ* hybridization (FISH) test suggested 29% positive c-MYC rearrangement. Positron emission tomography (PET) (**Figures 1H–O**) depicted multiple systemic enlarged lymph nodes coupled with abnormally high glucose metabolism, hepatosplenomegaly, significant myeloproliferative neoplasm, and infiltrated connective muscle tissue. This patient was evaluated by our surgeons, and they stated that the patient’s superficial lymph nodes were not big enough to yield a positive finding through a minimally invasive puncture biopsy. Moreover, performing an open-chest operation for biopsy was too traumatic, the localization was also challenging, and the patient’s parents refused it as well. Despite the lack of pathological biopsy, the diagnosis of B-cell lymphoma was considered more likely in combination with immunophenotyping analysis and FISH testing in the child. The patient did not show a significant decrease in trilineage cells at the beginning of the disease course, had a significantly higher LY% of peripheral blood, and did not show phagocytosis on bone marrow cell morphology after admission. In addition, based on the patient’s family history and genetic testing, it is not likely to be genetic HLH. Therefore, we considered HLH and lymphoma to be associated in this child.

**Table 1:** Routine blood and CSF investigations of the patient.

	Reference value	First day	Sixth day	Early stages of ANE	Discharge
WBC	(4–10) × 10^9^/L	3.86	1.23	1.03	13.39
LY	(20–40) %	36.8	49.6	10.8	21.6
NE	(50–75) × 10^9^/L	1.98	1.12	1.03	11.26
Hb	(110–140) g/L	92	75	77	107
Plt	(100–300) × 10^9^/L	198	143	139	433
Fib	(2–4) g/L	2.12	1.16	1.05	2.33
TG	(0–1.7) mmol/L	2.02	2.16	3.21	2.06
LDH	(172–382) U/L	668.2	789.0	1015.2	507.6
ALT	(5–35) U/L	36.2	39.8	114	43.2
SF	(22–640) pmol/L	688.4	1,102.5	2,472.1	672.1
sCD25	() pg/ml	–	1,668.8	1,356.4	Negative
IL-6	(0–5.9) pg/ml	–	138.2	–	Negative
TNF-α	(0.74–1.54) ng/ml	–	16.7	–	Negative
CSF protein	(120–600) mg/L	–	–	2,355	Negative
CSF IL-6	(0–5.9) pg/ml	–	–	98.6	Negative
CSF TNF-α	(0.74–1.54) ng/ml	–	–	15.3	Negative

WBC, white blood cell; LY%, percentage of lymphocytes; NE, neutrophil; Hb, hemoglobin; Plt, platelet; Fib, fibrinogen; TG, triglycerides; LDH, lactate dehydrogenase; SF, serum ferritin; sCD25, soluble CD25; IL-6, interleukin-6; CSF, cerebrospinal fluid; –, not done.

**Figure 3:**
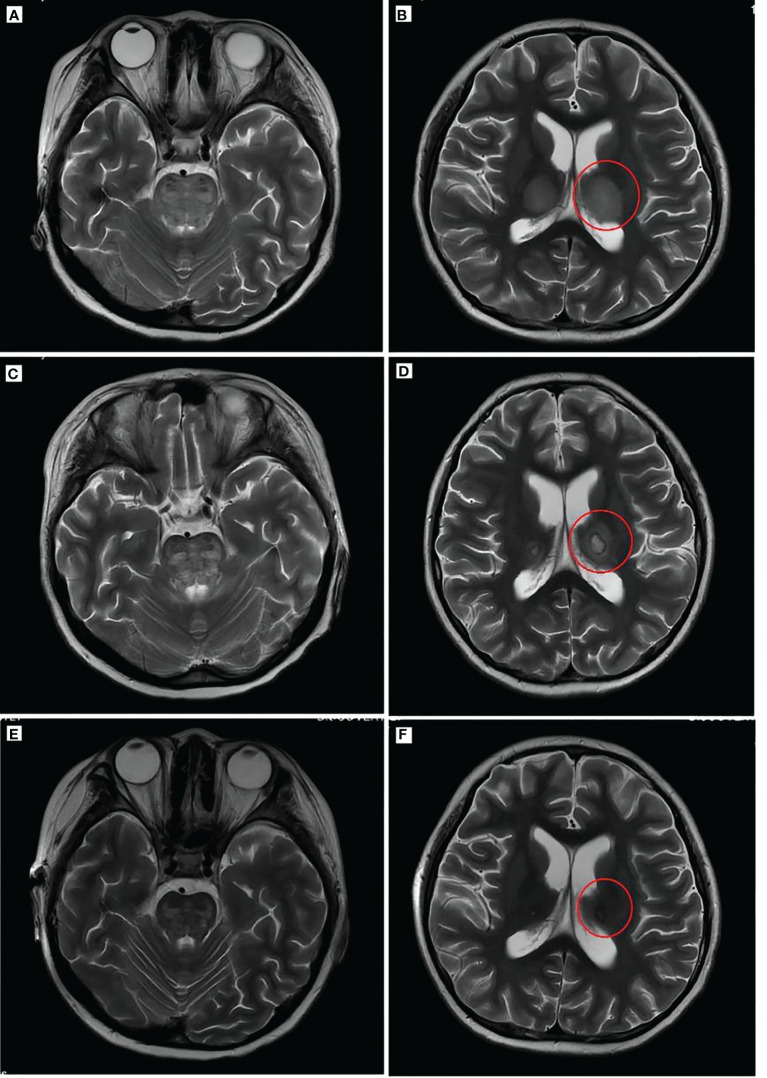
Brain imaging of the patient. T2WI **(A, B)** showed a high brainstem density and thalamus density (red circle). Brain T2WI **(C, D)** 2 weeks after ANE diagnosis indicated brainstem edema and typical “concentric circles” (red circles) within the thalamus. T2WI **(E, F)** after remission indicated the disappearance of edema and damage in the thalamus and brainstem. T2WI, T2-weighted MRI; PET, positron emission tomography; ANE, acute necrotizing encephalopathy.

During the first day of the CCHG-HLH-2018 chemotherapy regimen, the patient developed hyperthermia (40.6°C), vomiting, and decreased blood pressure (72/44 mmHg; 1 mmHg = 0.133 kPa), which improved through fluid infusion. The following morning, she also developed a rapid and progressive deterioration of consciousness, dysphagia, and dysarthria. On examination, the patient’s bilateral pupils were equally sized, but the light reflex was sluggish. The patient had limb weakness (R and L, lower, proximal predominant), and the right knee jerk reflex was ±. Blood investigations (**Table 1**) depicted the following results: decreased white blood cell count, 1.11 × 10^9^/L; absolute neutrophil count, 1.03 × 10^9^/L; hemoglobin, 77 g/L; platelet count, 139 × 10^9^/L; and fibrinogen, 1.05 g/L. She also had elevated triglycerides (3.21 mmol/L), lactate dehydrogenase (LDH) (1,015.2 U/L), glutamate transaminase (114 U/L), ferritin (2,472.1 pmol/L), and sCD25 (1,356.4 pg/ml). The cerebrospinal fluid (CSF) evaluation showed elevated opening pressure, normal cell count, and elevated protein level (2,355 mg/L), IL-6 (98.6 pg/ml), and TNF-α (15.3 ng/ml). CSF microbiology testing, culture, and “next-generation” sequencing (NGS) technology were negative. CT revealed low-density areas bilaterally in the thalami. MRI (**Figures 3A, B**) showed high-intensity brainstem and thalamus areas on T2-weighted images (T2WI), which indicated edema. After 2 weeks, the MRI (**Figures 3C, D**) still depicted symmetrical brain damage, and the thalamic damage revealed the typical “concentric circle” sign. Therefore, the final diagnosis was ANE.

**Figure 4:**
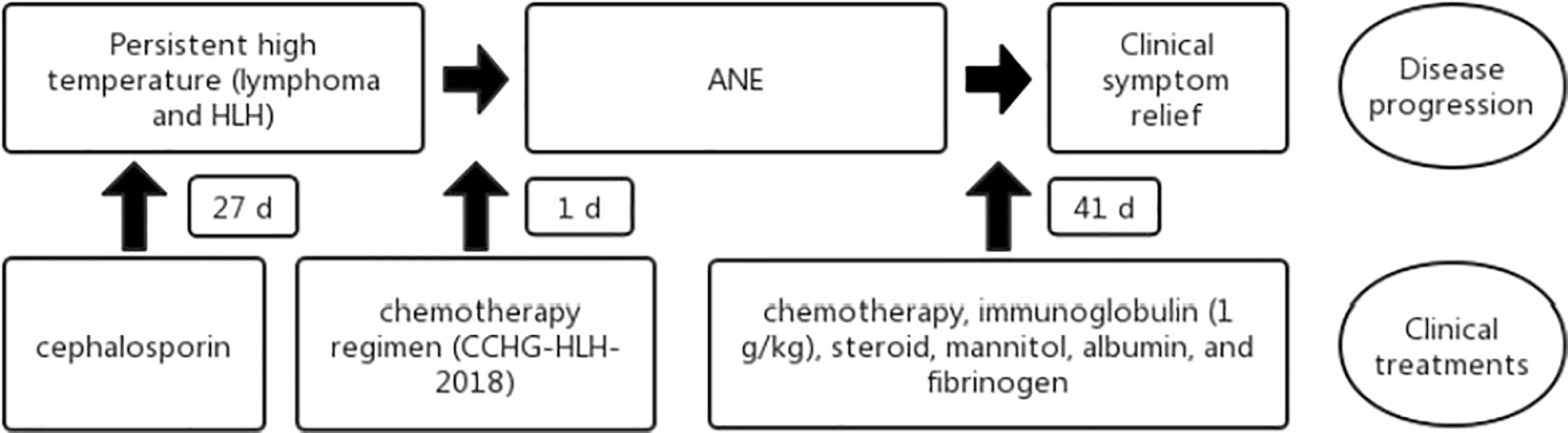
Flowchart of the patient disease progression and clinical treatments. HLH, hemophagocytic lymphohistiocytosis; ANE, acute necrotizing encephalopathy.

After 41 days of treatment, the child’s condition improved significantly. Primary treatments included chemotherapy, immunoglobulin (1 g/kg), steroid, mannitol, albumin, and fibrinogen. Additionally, the muscle strength of the limbs recovered more than before, all of which was V−, but dysarthria persisted with decreased sucking ability. Routine blood examinations (**Table 1**) showed the following: white blood cell count of 13.39 × 10^9^/L, absolute neutrophil count of 11.26 × 10^9^/L, hemoglobin of 107 g/L, and platelet count of 433 × 10^9^/L; decreased ferritin levels (672.1 pmol/L); normal fibrinogen and serum cytokine levels; and normal CSF, biochemical markers, and cytokines. Moreover, NK cell activity improved from the initial examination. Three weeks after the start of chemotherapy, bone marrow cell morphology (**Figure 2C**) indicated severe hypoplasia of bone marrow nucleated cells without phagocytic cells. Three days before discharge, bone marrow cell morphology (**Figure 2D**) revealed active proliferation of myeloid nucleated cells, normal morphology of all the cells, and no phagocytic cells. Necrotic lesions were still visible on brain MRI (**Figures 3E, F**) but significantly improved from the previous examination. PET showed that the original lymph nodes and soft tissue lesions significantly improved, and the glucose metabolism was significantly lower. The sequela of the child during follow-up 2 months after being discharged was only slow speech. Six months after being discharged, the patient was followed up and had fluent speech without neurological involvement. The patient and her guardians were satisfied with the treatment results, and the patient was followed up regularly, with no signs of disease recurrence. The parents also intended to have their child undergo allogeneic hematopoietic stem cell transplantation (allo-PBSCT). The patient's disease progression and treatment flow chart are shown in **Figure 4**.

## Discussion

HLH patients are characterized by persistent fever, bicytopenia, and hemophagocytosis in the bone marrow, liver, spleen, and lymph node tissues. HLH was first reported by Scott et al. in 1939. The incidence of the disease varies by age and race, with 1.2/1,000,000 in the European and Japanese populations and 1/100,000 in the US population (1, 7). The disease occurs in children and infants with a rapid onset, rapid progression, and poor prognosis. HLH is associated with a series of pathological changes due to the overactivation of the body’s macrophages, lymphocytes, and other immune cells. The common causes of HLH include genetic susceptibility, viral infections, autoimmune diseases, and malignancies, including lymphomas (2). The clinical manifestations, laboratory tests, and imaging examinations of this child meet the diagnostic criteria of HLH (8). Meanwhile, the case of HLH should be distinguished from genetic HLH. The peripheral blood of the child and her parents was analyzed by whole-exome sequencing, focusing on familial HLH (FHLH) and HLH associated with immunodeficiency disease (9), and no relevant abnormal mutations were found. There were no HLH patients in the child’s family. Therefore, it is not likely to be genetic HLH.

The pathological changes of neurological damage in HLH mainly include degeneration and necrosis due to lymphocyte and macrophage infiltration in the meningeal, cerebrovascular, and brain tissues. The most common brain injury imaging features of HLH are widespread brain atrophy, leukoaraiosis, and demyelinating encephalopathy. However, other specific findings include brain hemorrhage and edema (10, 11). Acute necrotizing encephalopathy associated with HLH is not uncommon (12–14). ANE was first proposed by Mizuguchi et al. in 1995 (5). Patients present acute viral infection, with further neurological manifestations such as twitching and consciousness disorder. These clinical symptoms are often accompanied by systemic inflammatory response syndrome (SIRS) manifestations such as shock, multiple organ dysfunction syndrome (MOD), and disseminated intravascular coagulation (DIC). ANE patients have a mortality rate of up to 30%, and the survivors often have moderate to severe disabilities. Brain imaging of ANE often reveals symmetric, multifocal CNS lesions, particularly in the thalamus and brainstem (4, 6, 15). A wide range of disorders should be considered in the differential diagnosis, including Leigh disease, Reye syndrome, Japanese encephalitis, hemorrhagic encephalitis, and acute disseminated encephalomyelitis. The patient presented with recurrent high fever and gradually developed dysphagia, dysarthria, consciousness disorder, and decreased muscle strength. Brain MRI showed symmetric thalamic and brainstem edema. Moreover, the typical “concentric circle” sign was seen on the repeated brain MRI 2 weeks later, consistent with the pathological changes of ANE (16), excluding hemorrhagic encephalitis, acute disseminated encephalomyelitis, and Japanese encephalitis. Leigh disease and Reye syndrome were not considered in children without hyperammonemia and lactic acidosis (17). After 1 day of chemotherapy, the child presented with neurological involvement, and ANE caused by the drugs could not be entirely excluded. However, ANE caused by chemotherapy drugs has not been reported and requires further research.

The pathogenesis of ANE is still unclear. A cytokine storm may play an essential role in the development of ANE. Cytokine storms are life-threatening systemic inflammatory syndromes involving elevated levels of circulating cytokines, immune cell hyperactivation, and secondary organ dysfunction, including the brain (18). The patient had elevated peripheral blood inflammatory factor levels prior to the onset of neurological involvement (IL-6 138.2 pg/ml, TNF-α 16.7 pg/ml). After the presentation of encephalopathy, her cerebrospinal fluid inflammatory factor levels were significantly elevated, and the brain MRI showed extensive cerebral edema (IL-6 98.6 pg/ml, TNF-α 15.3 pg/ml). IL-6 and TNF-α dominated the elevated inflammatory factors in the patient. There is evidence that high levels of IL-6 are neurotoxic (19). Moreover, elevated TNF-α levels can damage vascular endothelial cells, disrupt the blood–brain barrier (BBB), and induce myelin and oligodendroglia necrosis (20, 21). In a nutshell, peripheral inflammation may lead to BBB disruption, which induces CNS inflammatory response and further aggravates the destruction of the BBB, forming a vicious cycle that results in encephalopathy eventually (22, 23). Pensato et al. (24) defined CySE as follows: encephalopathy with acute or subacute onset, association with cytokine storm (as defined by Fajgenbaum et al.), and exclusion of other causes that might independently account for the severity of neurological manifestations. The clinical symptoms and brain imaging of the patient improved after steroid treatment. The cytokine levels in the serum and CSF were normalized, indicating proinflammatory cytokine overactivation and overexpression of SCKRs in HLH patients and could be associated with ANE development.

HLH treatment mainly includes remission therapy induction and etiological therapy. Induction remission therapy controls the cytokine storm to prevent HLH, and the etiological treatment can correct the underlying immunodeficiency in preventing HLH recurrence (1, 8). After a treatment course, this child’s clinical symptoms and related tests indicated HLH remission. ANE is extremely rare in HLH patients. The early application of steroids in ANE therapy to antagonize the cytokine storm is considered effective in clinical treatment (25, 26). In this case, steroid treatment was initiated early in ANE, and only dysarthria and decreased sucking ability remained after symptom resolution. Therefore, the early application of steroids in HLH patients could reduce ANE incidence and minimize the risk of death in established ANE patients. The child is now discharged from the hospital for 6 months with regular follow-up. There were no disease recurrence signs. Routine blood, lipid levels, liver function, ferritin, IL-6, and sCD25 were normal, and bone marrow cytology continues to indicate remission. The parents intend to have their child undergo allo-PBSCT.

The combination of LA-HLH with ANE could be associated with cytokine storm. Therefore, patients with HLH should be vigilant about developing ANE when presenting clinical manifestations of CNS involvement. Early steroid application to antagonize the cytokine storm has a better therapeutic effect on HLH. Moreover, it could also prevent the development of ANE and reduce the risk of death.

## Data availability statement

The original contributions presented in the study are included in the article/supplementary material. Further inquiries can be directed to the corresponding author.

## Author contributions

WS participated in the study design and writing of the manuscript. CF participated in clinical data collection and carried out the interpretation of the data. XZ participated in the data analysis, data interpretation, and manuscript writing. All authors read and approved the final manuscript.

## Acknowledgments

We would like to thank the patient for consenting to the publication of this case.

## Conflict of interest

The authors declare that the research was conducted in the absence of any commercial or financial relationships that could be construed as a potential conflict of interest.

## Publisher’s note

All claims expressed in this article are solely those of the authors and do not necessarily represent those of their affiliated organizations, or those of the publisher, the editors and the reviewers. Any product that may be evaluated in this article, or claim that may be made by its manufacturer, is not guaranteed or endorsed by the publisher.
